# *Paenibacillus polymyxa* Antagonism towards Fusarium: Identification and Optimisation of Antibiotic Production

**DOI:** 10.3390/toxins15020138

**Published:** 2023-02-09

**Authors:** Junjian Ran, Youzhi Wu, Bo Zhang, Yiwei Su, Ninghai Lu, Yongchao Li, Xinhong Liang, Haixu Zhou, Jianrong Shi

**Affiliations:** 1 School of Food Science, Henan Institute of Science and Technology, Xinxiang 453003, China; 2 School of Food and Drug, Shanghai Zhongqiao Vocational and Technology University, Shanghai 201514, China; 3 Institute of Food Quality and Safety, Jiangsu Academy of Agricultural Sciences, Nanjing 210014, China

**Keywords:** *Paenibacillus polymyxa*, antibiotic, deoxynivalenol, response surface methodology

## Abstract

An antibiotic produced by *Paenibacillus polymyxa* 7F1 was studied. The 7F1 strain was isolated from the rhizosphere of a wheat field. Response surface methodology was used to optimize the physicochemical parameters. The strain showed broad-spectrum activity against several plant pathogens. Identification of the strain was realized based on 16s rRNA gene and *gyrB* gene sequencing. The antibiotic was optimized by one-factor-at-a-time (OFAT) and response surface methodology (RSM) approaches. The suitable antibiotic production conditions were optimized using the one-factor-at-a-time method. The individual and interaction effects of three independent variables: culture temperature, initial pH, and culture time, were optimized by Box-Behnken design. The 16SrRNA gene sequence (1239 nucleotides) and *gyrB* gene (1111 nucleotides) were determined for strain 7F1 and shared the highest identities to those of *Paenibacillus polymyxa*. The results showed the optimal fermentation conditions for antibiotics produced by *Paenibacillus polymyxa* 7F1 were a culture temperature of 38 °C, initial pH of 8.0, and culture time of 8 h. The antibiotics produced by *Paenibacillus polymyxa* 7F1 include lipopeptides such as iturin A and surfactin. The results provide a theoretical basis for the development of bacteriostatic biological agents and the control of mycotoxins.

## 1. Introduction

*Fusarium* head blight (FHB) is a severe disease of wheat, corn, barley, and other grain and occurs in all regions worldwide. Researchers have found that 17 species of *Fusarium* are associated with the symptoms of FHB [1]. The following *Fusariums* are the main causal species of FHB: *Fusarium graminearum*, *Fusarium avenaceum*, *Microdochium nivale, Fusarium culmorum,* and *Fusarium poae*, especially the *Fusarium graminearum* species complex [2,3,4]. These pathogens reduce wheat yield and quality by infecting wheat spikes. In addition, FHB pathogens produce substances that are toxic to humans and other animals, such as deoxynivalenol (DON) and nivalenol (NIV) [5]. The incidence of food poisoning and mycotoxin presence vary considerably from year to year and are influenced by environmental growing conditions, local agronomic systems, and interactions between the two. Since *Fusarium* can survive on saprotic crop remnants of host plants such as maize and wheat, no-tillage or reduced-tillage regimes favor FHB infection.

Over the past few decades, there has been a growing desire to reduce the use of harmful chemical fungicides and control plant diseases by other means. This desire has led scientists to study more and more microbial agents to control plant diseases. It has been shown that certain microorganisms can inhibit the growth of *Fusarium* wheat in previous studies, and these microorganisms do not pollute the environment, reducing the use and dependence on chemical pesticides, thereby slowing down the development of chemical pesticide resistance to pathogens. [6]. Several bacteria and fungi have been found to inhibit the growth of *Fusarium graminearum* [7]. Among the antimicrobial agents identified, *Bacillus* is the most compelling antibiotic-producing strain, and it has more advantages than other biocontrol microorganisms due to its inherent endospore formation and resistance to extreme conditions. *Bacillus* strains were demonstrated by in vitro antibacterial [8], reducing the severity of disease caused by in situ destructions of spikelet infection [9] and identification of lipopeptides [10]. In the study of antibacterial mechanisms, the production of antifungal compounds is considered the primary mode of action against bacteria.

Response surface methodology (RSM) is a method to simulate absolute limit-state surfaces through a series of deterministic tests with multiple variables. The main advantage of RSM is to fit the complex unknown function relationship in a small area with a simple first or second-order polynomial model, which is simple to calculate and can be analyzed continuously at every level of the experiment. It is widely used in optimizing the extraction process variables [11,12,13,14,15,16]. Box-Behnken design has three levels and needs fewer experiments. Their operating cost is lower than that of central composite structures with the same number of factors, and they are widely used by researchers [17,18].

Several conditions, such as initial pH, culture temperature, and culture time, must be optimized to maximize levels of antibiotic produced by *Paenibacillus polymyxa* 7F1. In this study, a strain of *Paenibacillus polymyxa* with an antagonistic effect was identified, and the conditions for producing the antibiotic were optimized by OFAT and RSM methods.

## 2. Results

### 2.1. Identification of Antagonistic Bacteria 7F1

Strain 7F1 was isolated and purified from the rhizosphere of a wheat field in Nanjing and showed a robust antagonistic effect on the *Fusarium graminearum* GZ3639 strain. Strain 7F1 was identified as a Gram-positive, catalase-positive, facultatively anaerobic, milky rod-shaped bacterium with an oval endospore center by physiological and biochemical tests. Table 1 showed that strain 7F1 could not use inositol and citrate as carbohydrate sources to produce gas and acid. The salt tolerance test results showed that strain 7F1 was not able to grow above 4% NaCl concentration in (Luria-Bertani) LB medium.

**Figure 1:**
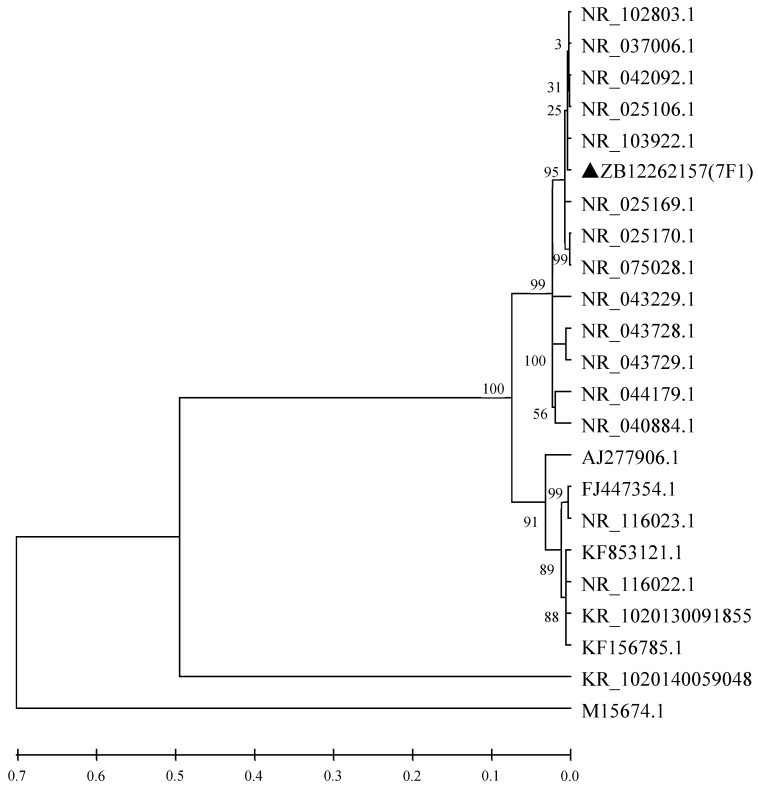
Phylogenetic tree based on 16 S rRNA sequence of strain 7F1. NR_102803.1: *Paenibacillus polymyxa*; NR_042092.1: *Paenibacillus peoriae*; NR_037006.1: *Paenibacillus polymyxa*; NR_103922.1: *Paenibacillus polymyxa*; ▲ZB12262157 (7F1): *Paenibacillus polymyxa*; NR_025169.1: *Paenibacillus kribbensis*; NR_025170.1: *Paenibacillus rerrae*; NR_075028.1: *Paenibacillus rerrae*; NR_044179.1: *Paenibacillus provencensis*; NR_040884.1: *Paenibacillus illinoisensis*; NR_043728.1: *Paenibacillus forsythiae*; NR_043229.1: *Paenibacillus woosongensis*; NR_043729.1: *Paenibacillus sabinae*; KR 1020140059048: *Bacillus amyloliquefaciens*; KR 1020130091855: *Bacillus amyloliquefaciens*; NR_116022.1: *Bacillus amyloliquefaciens*; KF 156785.1: *Bacillus amyloliquefaciens*; M 15674.1: *Bacillus amyloliquefaciens*; FJ 447354.1: *Bacillus licheniformis*; NR_116023.1: *Bacillus licheniformis*; AJ 277906.1: *Bacillus subtilis*; KF 853121.1: *Bacillus subtilis.* Numbers behind each sequence are NCBI Genbank NO.

**Figure 2:**
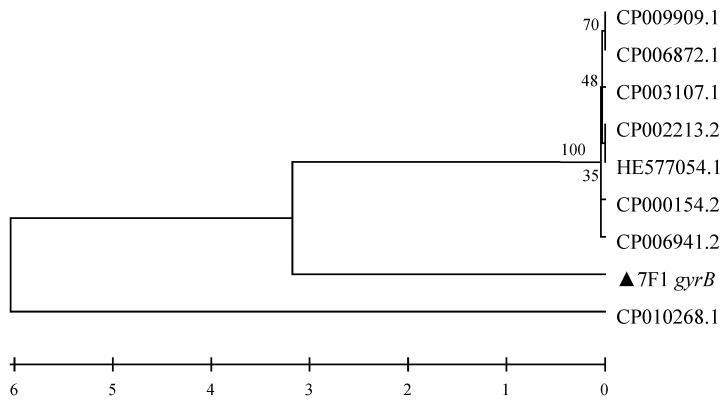
Phylogenetic tree based on *gyrB* sequence of strain 7F1. CP009909.1: *Paenibacillus polymyxa*; CP006872.1: *Paenibacillus polymyxa*; CP003107.1: *Paenibacillus polymyxa*; CP002213.2: *Paenibacillus polymyxa*; HE577054.4: *Paenibacillus polymyxa*; CP000154.2: *Paenibacillus polymyxa*; CP006941.2: *Paenibacillus terrae*; ▲7F1 *gyrB*: *Paenibacillus polymyxa*; CP010268.1: *Paenibacillus polymyxa*. Numbers behind each sequence are NCBI Genbank NO.

From a 1239 bp region of the 16s rRNA sequence and an 1111 bp region of the *gyrB* sequence of 7F1 (GeneBank Accession NO. KP410736) compared with that of known species of microorganisms, it was confirmed that 7F1 shared 99% sequence similarity with that of *Paenibacillus polymyxa* (Figure 1 and Figure 2). Therefore, strain 7F1 was identified as *Paenibacillus polymyxa*.

### 2.2. Antifungal Activity and Culture Conditions for Antibiotic

**Figure 3:**
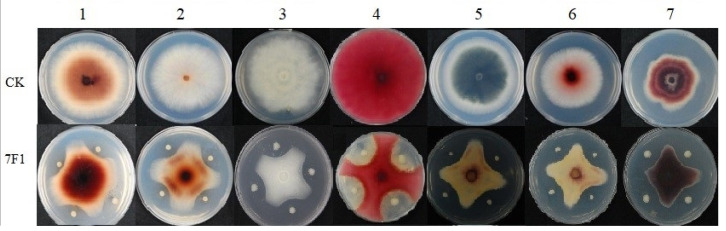
Antifungal activity of *Paenibacillus polymyxa* 7F1 against seven pathogenic fungi. 1. Fusarium equiseti (CGMCC 3.6911), 2. Fusarium verticillioide (CGMCC 3.7987), 3. Fusarium semitectum (CGMCC 3.6808), 4. Fusarium graminearum GZ3639, 5. Colletotrichum gloeosporioides (IVFCAAS PP08050601), 6. Fusarium proliferatum (CGMCC 3.4741), 7. Fusarium oxysporum (CGMCC 3.6855).

*Paenibacillus polymyxa* 7F1 showed high antifungal activity against all fungi strains tested in this study. Figure 3 shows the antifungal activity of *Paenibacillus polymyxa* 7F1 against seven pathogenic fungi.

**Figure 4:**
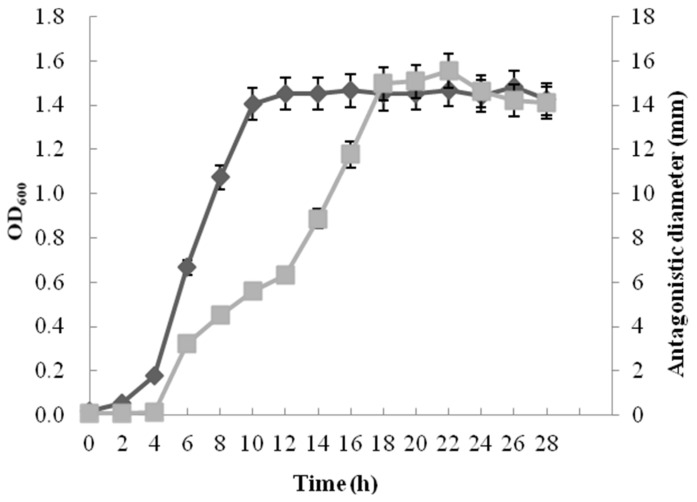
Growth curve (dark gray squares) and antimicrobial activity (light gray squares) of *Paenibacillus polymyxa* 7F1.

The results of the time course experiment revealed that the antibiotic produced by *Paenibacillus polymyxa* 7F1 was growth dependent. At the culture temperature of 37 °C, the antifungal activity and OD_600_ value increased with the extension of culture time, and the isolate had the highest antifungal activity and OD_600_ value at 28 h of incubation with an initial pH of 7.0 and temperature of 37 °C (Figure 4).

### 2.3. Model Fitting and ANOVA

The response values for each set of the variable combinations are shown in Table 2, and the statistical analysis shows that the response values are most consistent with the second-order polynomial model, which is then adopted. The models had satisfactory levels of adequacy (R^2^). Antagonistic diameter (mm) varied from 8.37 to 16.46 (Table 2). All models for antibiotics are shown in Table 3 and Table 4 and found to be significant. The regression analysis of the optimization study indicated that the model terms *X*_1_, *X*_2_, *X*_3_, *X*_1_*X*_2_, *X*_2_*X*_3_, *X*_1_^2^, and *X*_3_^2^ were significant (*p* < 0.05), the *X*_1_*X*_2_, *X*_2_^2^ were not significant (*p* > 0.05). These results indicate that the interactions between culture temperature, initial pH, and culture time are directly related to antibiotic production. The coefficient of determination R^2^ was 0.9945, indicating that 99.45% of the sample variation was attributable to variables, and only 1% of the total variance could not be explained by the model. Regression models with R^2^ values greater than 0.9 are considered to have a high correlation [12]. Therefore, the R^2^ value reflected a perfect fit between the observed and predicted responses. The adjusted determination coefficient (R^2^ Adjusted = 98.47%) also satisfactorily confirmed the significance of the model. Regression analysis of experimental data was carried out to fit the mathematical model to determine the optimal region of the studied response. The predicted response *Y* of each response yield could be expressed in coded values using the following second-order polynomial equation:

*Y* = −324.92 + 12.93*X*_1_ + 16.10*X*_2_ + 5.88*X*_3_ − 0.25*X*_1_*X*_2_ − 0.04X_1_X_3_ − 0.24*X*_2_*X*_3_ − 0.14*X*_1_^2^ − 0.24*X*_2_^2^ − 0.15*X*_3_^2^ (1)

where *Y* is the antagonistic diameter, and *X*_1_, *X*_2_, and *X*_3_ are the variables for culture temperature, initial pH, and culture time, respectively.

In general, exploration and optimization of fitted response surfaces can produce poor or misleading results unless the model exhibits a good fit, so it is critical to check the adequacy of the model [19]. The F-ratio in this table is the ratio of the mean square error to the pure error obtained from the repetition of the design center. The significance of the F-value depends on the number of degrees of freedom (DF) in the model and is shown in the *p*-value column (95% confidence level). Therefore, the column below 0.05 has a significant effect [20].

Table 4 listed the analysis of variance (ANOVA) for the fitted quadratic polynomial model of antagonistic diameter. The lack of fit was an essential term in the functional relationship between factors and response variables of regression models. As shown in Table 4, the *p*-value of the lack of fit was 0.32, meaning that it’s not significant relative to the pure error and suggesting that the model equation is sufficient to predict the antagonistic diameter for any combination of variable values. The *p*-value was used as a tool to check the significance of each coefficient and the strength of the interaction between each independent variable [21]. The higher it is, the better correlation between the observed values and predicted values [22].

### 2.4. Analysis of Response Surfaces

Since the models showed a lack of fit is not significant, the response values could be adequately explained by the regression equation. The regression models allowed the prediction of the effects of the three parameters on antagonistic diameter. The relationship between independent and dependent variables was explained by establishing a three-dimensional curved surface and a two-dimensional contour plot generated of the response surface. Based on coded data, when different factors interact with each other, the antibacterial zone diameter of the antibiotic has a saddle point, which is the maximum value when factors interact with each other.

**Figure 5:**
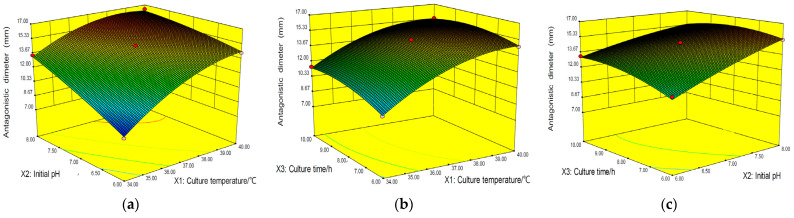
The effects of three factors on antagonistic diameter by response surface model plot. (**a**) Interaction between culture temperature and initial pH; (**b**) Interaction between culture temperature and culture time; (**c**) Interaction between initial pH and culture time.

It’s indicated from Figure 5a that the maximum antagonistic diameter at optimum culture temperature and a high initial pH value. Hence the effect of initial pH was dependent on culture temperature. Figure 5b depicts an increase in culture temperature and culture time resulting in the increased antagonistic diameter. However, the trend is reversed with further increases in culture temperature and culture time. A similar trend was observed in the initial pH and culture time interaction (Figure 5c). Hence increasing the initial pH would increase the antagonistic diameter at optimum culture time and temperature.

### 2.5. Optimization of the Models

The primary goal of the optimization process was to maximize response value. The results for the three analyzed parameters and the maximum predicted and experimental value is given in Table 5. Additional experiments using the predicted optimum conditions for antagonistic diameter were carried out: culture temperature of 38.83 °C, initial pH of 8.00, culture time of 7.87 h, and the model predicted a maximum response of 16.46 mm. Experiment rechecking was performed using these modified optimal conditions to ensure the predicted result did not bias the practical value: culture temperature of 38 °C, initial pH of 8, culture time of 8 h, and the mean value of 16.38 ± 0.084 mm (*n* = 5) was attained, there was a significant agreement with the predicted value (*p* > 0.05) to verify the validity of the RSM model. The above results showed that the response model could fully reflect the expected optimization results, and the model was satisfactory and accurate.

### 2.6. Identification of Antibiotic

**Figure 6:**
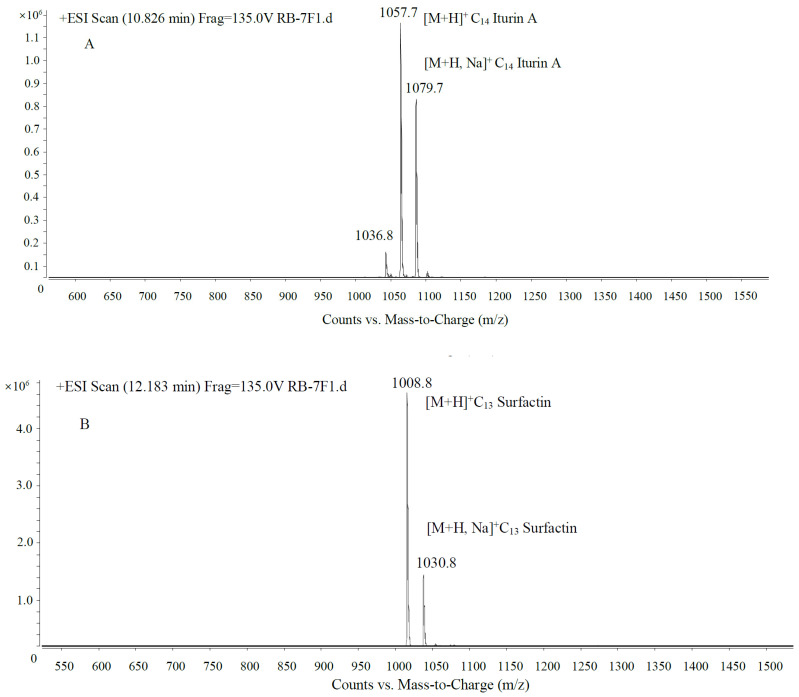

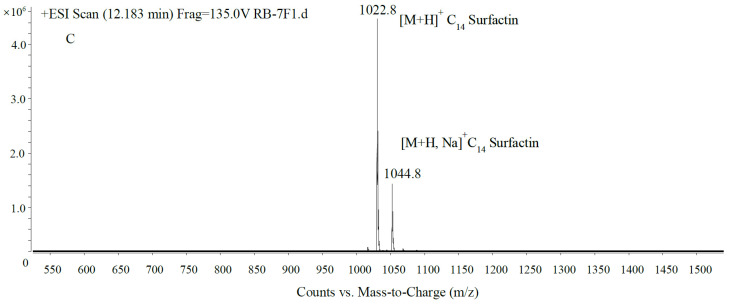
Mass spectrum of active peaks ((**A**). 1057.7, (**B**). 1008.8, and (**C**). 1022.8) detected by HPLC.

Antibiotics produced by *Paenibacillus polymyxa* 7F1 were separated using HPLC-MS. Three peaks were analyzed to confirm the active peak. The molecular weight of the chemicals was elucidated by LC-ESI mass spectrometry [23,24]. Data from the positive ion mass spectrum analysis showed that antibiotics produced by *Paenibacillus polymyxa* 7F1 contain a variety of compounds, three peaks showed good antagonistic effect among them, the three active peaks were analyzed by secondary mass spectrometry, [M+H]^+^ ion peaks were 1057.7, 1008.8, and 1022.8, respectively (Figure 6). They corresponded to C_14_ iturin A, C_13_ surfactin, and C_14_ surfactin, respectively.

## 3. Discussion

FHB has always been an important disease threatening the yield and quality of the grain, especially in 2010 and 2012, which caused a large number of grain production reductions [25]. There has been no good solution to the control of FHB, such as the long-term use of carbendazim and other chemical pesticides, and there is no variety completely immune to FHB so far, so an alternative method to control FHB is urgently needed [26]. Biological control refers to the use of natural organisms or their metabolites to control plant diseases, which mainly includes three categories, namely, treating diseases with bacteria, treating insects with insects, and treating insects with bacteria [6]. Nowadays, resistance breeding and chemical control have made some achievements in controlling FHB. With the rapid development of ecological pathology, it has become the focus of attention to find biological control measures that give attention to disease prevention, yield increase, and environmentally friendly and sustainable development from the perspective of microorganisms [7]. It has been a very active and promising development field to control many kinds of plant diseases from beneficial microorganisms and achieve comprehensive control of plant diseases. Among them, the use of biocontrol *bacillus* to control plant diseases has been a developing trend in recent years. *Bacillus* has strong stress resistance, is harmless to people and animals, and does not pollute the environment [27]. It is a good biocontrol bacterium and can produce a variety of antagonistic substances during fermentation culture, including bacteriocin, antibiotics, antibacterial proteins, degrading enzymes, and so on.

In this study, *Paenibacillus polymyxa* 7F1 was isolated from wheat rhizosphere soil, and the antibiotic produced by *Paenibacillus polymyxa* 7F1 had a good inhibitory effect on *Fusarium graminearum*. Optimization of cultivation conditions was investigated through the OFAT and RSM applications. Based on the OFAT, RSM was used to estimate and optimize the experiential variables-culture temperature (°C), initial pH, and culture time (h). To gain a better understanding of the effects of variables on the antibiotic, the predicted model was presented as two-dimensional surface plots [28,29]. All the independent variables, *X*_1_, *X*_2_, *X*_3_, *X*_1_*X*_2_, *X*_2_*X*_3_, *X*_1_^2^, and *X*_3_^2^ culture temperature, initial pH, culture time, the interaction of culture temperature and initial pH, the interaction of initial pH and culture time, quadratic of culture temperature and quadratic of culture time had significant effects on the response value. A high correlation of the quadratic polynomial mathematical model was gained and could be a highly produced antibiotic by *Paenibacillus polymyxa* 7F1. The optimal conditions for antibiotics were determined as follows: culture temperature of 38 °C, initial pH of 8, and culture time of 8 h. The capacity of the model equation to predict the optimal response value is proved by the test under the optimal conditions [30,31]. Under these conditions, the experienced antagonistic parameter was 16.38 ± 0.084 mm, which agreed closely with the predicted value.

The antibacterial substances produced by *Bacillus* mainly include lipopeptides, antibacterial proteins, polyketones, etc., according to reports [32,33], among which lipopeptide antibiotics such as Fengycin, Iturin, and Surfactin synthesized by non-ribosomal pathway [34] are the main antibiotics produced by *Bacillus* fermentation and play an important role in inhibiting fungal diseases. Ongena et al. found that esubtilisin can destroy the cell membrane of yeast cells, make potassium ions and other important substances permeate, and cause the death of yeast cells [35]. Antifungal subtilisin produced by *Bacillus subtilis* (an important active substance of the esubtilisin family) can inhibit the growth of various yeasts, but the best effect is to inhibit *Aspergillus flavus* (*Aspergillus* spp.) [36]. Fenyuan can affect the surface tension of fungal cell membranes, lead to the formation of micropores, the leakage of potassium ions and other important ions, and cause cell death, but it has no significant effect on the morphology and cell structure of *Fusarium oxysporum* [37]. Zhao et al. believe that *B. subtilis* SG6 produced progenin and surfactant in the inhibition of *Fusarium graminearum* and played a major role in the growth process [38]. The results of mass spectrometry indicated that the antibiotic produced by *Paenibacillus polymyxa* 7F1 contained lipopeptides such as iturin A and surfactin. The identification results in this paper are consistent with those in the literature.

Among the biological control agents against plant diseases, the effect of antagonistic bacteria is more prominent than other control materials. The reasons are analyzed as follows: Firstly, some studies have pointed out that biological control mainly promotes the growth and development of host plants to form systematic resistance through antibiotic action, competition, increasing the solubility of inorganic nutrients, and inducing the host to produce disease resistance [39]. When antagonistic bacteria play a biocontrol role, several biocontrol mechanisms often act at the same time, which makes it difficult for pathogenic bacteria to produce drug resistance. Secondly, the source of antagonistic bacteria is mostly farmland, which has the same ecological adaptability as pathogenic bacteria, and the colonization effect is better [40]. Moreover, bacteria multiply relatively rapidly, and it is easier to fight against pathogenic bacteria to achieve the purpose of disease prevention and control, and the prevention and control cycle is longer than other microorganisms. Thirdly, bacteria have the characteristics of a short metabolic cycle, and rapid reproduction and passage [10], so antagonistic substances are produced at an alarming rate. In field experiments and popularization, the synergistic effect of living bacteria and their antagonistic metabolites can greatly improve the potential of antagonistic bacteria to play a biocontrol role and, at the same time, save time and control costs. Finally, the use of beneficial microorganisms for biological control is more friendly to the ecological environment and beneficial to ecological balance [41]. The antagonistic substances produced by it have strong specificity, so they only target some pathogenic bacteria, do not react with other beneficial microorganisms, are generally harmless to the human body, and pose little or no harm to the ecosystem. Therefore, using antagonistic microorganisms to replace traditional chemical pesticides has great potential and market application prospects.

## 4. Materials and Methods

### 4.1. Strain and Culture Conditions

The antagonistic bacterium 7F1 was a wild-type strain initially isolated and identified from the rhizosphere soil of wheat infected with FHB and stored at −70 °C in 20% glycerol in the laboratory. *Fusarium graminearum* GZ3639 was supplied by the Department of Agriculture, Agriculture Research Service, Peoria, IL, USA.

The frozen strains were taken out and streaked onto Luria-Bertani agar, pH 7.0. After a 24 h incubation at 30 °C, strains were removed from the surface of the medium and inoculated into 100 mL of Luria-Bertani liquid culture (10 g tryptone/L; 5 g yeast extract/L; 10 g NaCl/L; the pH was adjusted to 7.0) using inoculation rings in a 250 mL shake flask and cultivated at 37 °C and 170 r/min for 24 h as a pre-culture [4]. The bacterial concentration of pre-culture reached 10^8^–10^9^ CFU/mL. 10 mL pre-culture medium was placed in 100 mL Luria-Bertani liquid medium in a 250 mL Erlenmeyer flask and cultured under the same conditions to produce antibacterial substances.

### 4.2. Identification of Antagonistic Bacteria 7F1

Polymerase chain reaction (PCR) amplification of the bacterial 16S rRNA gene and partial fragments of the *gyrB* gene was used to identify the antagonistic bacteria 7F1 [4]. The reaction system was 10 × Taq buffer (Mg^2+^ free) 5.0 μL, MgCl_2_ (25 mmol/L) 3.0 μL, dNTP mixer (10 mmol/L) 1.0 μL, primer 27F 1.5 μL, primer 1492R 1.5 μL, template (DNA) 0.5 μL, rTaq (5 U/μL) 0.3 μL, and ddH_2_O 37 μL. The 16S rRNA primers were 27F (5′AGAGTTTGATCMTGGCTCAG3′) and 1492R (5′GGTTACCTTGTTACGACT T3′). The PCR program was 98 °C for 2 min followed by 30 cycles of 98 °C for 15 s, 55 °C for 30 s, and 72 °C for 1.5 min, with a final 10 min extension at 72 °C. The *gyrB* primers were UP-1 (5′GAAGTCATCATGACCGTTCTGCA3′) and UP-2Sr (5′AGCAGGGTACGGATGTGCGAGCC3′). The PCR program was 95 °C for 5 min followed by 30 cycles of 95 °C for 1 min, 60 °C for 1 min, and 72 °C for 2 min, with a final 10 min extension at 72 °C. Following amplification, the PCR product was sequenced with ABI prism 377 DNA Sequencer (PE Applied Biosystems, Waltham, MA, USA) and compared with sequences in NCBI.

### 4.3. Extraction of Antibiotic

A total of 100 mL of fermentation medium was taken and centrifuged at 10,000 rpm for 20 min. The supernatant was slowly added to an ice bath with ammonium sulfate to 60% saturation and placed in a refrigerator at 4 °C overnight, and then the supernatant was centrifuged at 10,000 rpm for 20 min. The supernatant was discarded, and the precipitation was suspended with a phosphate buffer solution (pH 6.8). The suspension was put into a dialysis bag (8000–14,000 D of interception), fully desalted at 4 °C, and freeze-dried; the antibiotic was obtained and dissolved in 1 mL sterile water until use [37,38].

### 4.4. Antifungal Test

The antifungal activity was determined by the agar well diffusion method [42]. Potato glucose Agar (PDA) medium was used for antifungal experiments. The medium was prepared with 200 g potato, 20 g glucose, and 15 g Agar dissolved in 1000 mL distilled water. PDA plates were prepared to contain *Fusarium graminearum* GZ3639 spore 10^4^ per mL. In the plates, wells of 6.0 mm diameter were cut using a sterile steel borer. Then 50 μL of the antibiotic were added into the wells, respectively, followed by aerobic incubation at 28 °C for 48 h. The same volume of PBS (pH 6.8) was used as a control. The antibacterial effect was judged by observing the antibacterial zone, and the diameter of the circle’s antibacterial zone relative to each index was measured with a caliper. Other test strains fungi: *Fusarium equiseti* (CGMCC 3.6911)*, Fusarium verticillioide* (CGMCC 3.7987)*, Fusarium semitectum* (CGMCC 3.6808)*. Colletotrichum gloeosporioides* (IVFCAAS PP08050601)*, Fusarium proliferatum* (CGMCC 3.4741)*, Fusarium oxysporum* (CGMCC 3.6855).

### 4.5. Selection of the Suitable Conditions for Antibiotics by OFAT Approach

Initial screening of the appropriate conditions for antibiotics was performed by the OFAT method [15]. Five conditions: culture time, initial pH, culture temperature, shaking speed, and bottled fluid volume, were investigated. The incubation step was described as before. Samples were collected and tested for their antifungal activity.

### 4.6. Optimization of Antibiotic by RSM

RSM was used to optimize antibiotics produced by *Paenibacillus polymyxa* 7F1. The following three variables were selected using the Box-Behnken design (a three-factor three-level response surface analysis method): culture temperature (*X*_1_), initial pH (*X*_2_), and culture time (*X*_3_). The level of selected factors was determined by a single-factor experiment. These three single factors were coded at three levels (−1, 0, 1), which resulted in an experimental design of 17 experimental points. Experimental data were analyzed to fit a second-order polynomial model containing the coefficient of linear, quadratic, and two factors interaction effects. Design Expert 8.0.5 (Stat-Ease Inc., Minneapolis, MN, USA) was used for designing experiments and statistical data analysis (ANOVA) [43]. Outliers were identified and excluded when necessary in describing the relevant models. The model equation of response (*Y*) of the independent variables by the Design Expert (8.05b) software was:

(2) $$Y=\beta_{0}+\sum_{i=1}^{n}\beta_{i}X_{i}+\sum_{i=1}^{n}\beta_{ii}X_{i}^{2}+\sum_{i=1}^{n}\sum_{j>1}^{n}\beta_{ij}X_{i}X_{j}$$

where *Y* was the dependent variable, *β*_0_ was the constant coefficient, *β_i_* was the linear coefficient (primary effect), *β_ii_* was the quadratic coefficient, *β_ij_* was the two factors interaction coefficient, *n* was the number of factors studied and optimized in the experiment; *X_i_*, *X_j_* was the encoded independent variables. The fitting quality of the model was evaluated by determination coefficient (R^2^) and ANOVA. The establishment of the response surface and contour plot was obtained by quadratic multinomial fitting equation obtained through regression analysis, the two independent variables were kept at the constant values corresponding to the stable points, and the other two variables were changed. The response values predicted by the process equations were compared to the actual measured response values. The % error between the predicted and actual values was obtained. Optimization of process parameters was conducted using the Design Expert 8.0.5 software by combining numerical simulation coupled with the desirability function.

### 4.7. Identification of Antibiotic

The accurate molecular mass and structure of the purified antibiotic were determined by employing a reversed-phase capillary LC directly coupled to the mass spectrometer (LC-MS) [11]. The antibiotic obtained from 4.3 was filtered through a 0.22 μm water membrane and determined by high-performance liquid chromatography directly coupled to the mass spectrometer (HPLC-MS) (TRIPLE QUAD 3500, AB SCIEX, Framingham, MA, USA). Diamonsil C18 reversed-phase column (250 mm × 4.6 mm, 5 μm) (Dikema Technology Inc., Beijing, China) was used. The mobile phase consisting of 0.1% trifluoroacetic acid aqueous solution and acetonitrile, was programmed at the flow rate of 0.6 mL/min. The sample volume injected was 20 μL. Antibiotics were eluted in a gradient system: 0–15 min, linear gradient 30–45% (B), 15–40 min, linear gradient 45–55% (B). The antibiotics were monitored at a wavelength of 280 nm. The MS analysis was conducted by electrospray ionization in positive ion mode.

### 4.8. Statistical Analysis

The resulting data are shown as the mean ± standard (*n* = 5), and the SPSS 18.0 software was used to analyze the results (Munich, Bavaria, Germany). ANOVA was used for statistical analysis, with α = 0.05 as the significance level.

## Figures and Tables

**Table 1:** Biochemical characteristics of *Paenibacillus polymyxa* 7F1.

Certified Variety	7F1
Catalase test	+
pH 7.0	+
Amylase test	+
Gelatin hydrolysis	+
Citrate utilization	−
Voges-Proskauer test	+
Indole production	−
1% NaCl	+
4% NaCl	+
8% NaCl	−

+ Positive; − Negative.

**Table 2:** Response surface design and corresponding response values.

Trail NO.	Coded Value			Real Value			*Y* Observed/(mm)	*Y* Predicted/(mm)	*Y* Residual/(mm)
	*X*_1_	*X*_2_	*X*_3_	*X*_1_/°C	*X*_2_	*X*_3_/h			
1	1	0	−1	40	7	6	14.63	14.74	−0.11
2	0	1	1	37	8	10	15.16	15.43	−0.27
3	0	0	0	37	7	8	14.45	14.63	−0.18
4	1	−1	0	40	6	8	14.35	14.51	−0.16
5	0	0	0	37	7	8	14.84	14.63	0.21
6	−1	0	−1	34	7	6	9.73	9.80	−0.071
7	0	1	−1	37	8	6	15.27	15.36	−0.089
8	−1	0	1	34	7	10	11.42	11.31	0.11
9	1	0	1	40	7	10	15.36	15.29	0.071
10	0	0	0	37	7	8	14.61	14.63	−0.023
11	1	1	0	40	8	8	16.46	16.26	0.20
12	−1	1	0	34	8	8	13.45	13.29	0.16
13	0	−1	−1	37	6	6	11.43	11.16	0.27
14	−1	−1	0	34	6	8	8.37	8.57	−0.20
15	0	−1	1	37	6	10	13.24	13.15	0.089

**Table 3:** Significance test of regression equation coefficient.

Source	Sum of Squares	df	Mean Square	F Value	*p*-Value
Model	72.85	9	8.09	101.15	<0.0001
Residual	0.40	5	0.080		
Lack of Fit	0.32	3	0.11	2.80	0.2739
Pure Error	0.077	2	0.038		
Cor Total	73.25	14			

C.V. = 2.09, R^2^ = 0.9945.

**Table 4:** ANOVA for response surface model.

Factor	Coefficient Estimate	df	Standard Error	F Value	*p*-Value
Intercept	16.43	1.00	0.16		
*X*_1_	2.23	1.00	0.10	496.62	<0.0001
*X*_2_	1.62	1.00	0.10	261.98	<0.0001
*X*_3_	0.51	1.00	0.10	26.52	0.0036
*X*_1_ *X*_2_	−0.74	1.00	0.14	27.56	0.0033
*X*_1_ *X*_3_	−0.24	1.00	0.14	2.88	0.1505
*X*_2_ *X*_3_	−0.48	1.00	0.14	11.52	0.0194
*X*_1_^2^	−1.23	1.00	0.15	70.14	0.0004
*X*_2_^2^	−0.24	1.00	0.15	2.72	0.1598
*X*_3_^2^	−0.62	1.00	0.15	17.48	0.0087

**Table 5:** Optimum conditions and the predicted and experimental values of response at the optimum conditions.

Name	Culture Temperature/°C	Initial pH	Culture Time/h	Antagonistic Diameter/mm
Optimum conditions	38.83	8.00	7.87	16.46 (predicted)
Modified conditions	38	8	8	16.38 ± 0.084 (Actual)

## Data Availability

The data presented in this study are available on request from the corresponding author. The data are not publicly available.
